# Australia needs to increase testing to achieve hepatitis C elimination

**DOI:** 10.5694/mja2.50544

**Published:** 2020-03-13

**Authors:** Nick Scott, Rachel Sacks‐Davis, Amanda J Wade, Mark Stoove, Alisa Pedrana, Joseph S Doyle, Alexander J Thompson, David P Wilson, Margaret E Hellard

**Affiliations:** ^1^ Burnet Institute Melbourne VIC; ^2^ St Vincent's Hospital Melbourne VIC

**Keywords:** Hepatitis, viral, Epidemiology

## Abstract

**Objectives:**

To assess progress in Australia toward the 2030 WHO hepatitis C elimination targets two years after the introduction of highly effective direct‐acting antiviral (DAA) treatments.

**Design:**

Analysis of quarterly data on government‐subsidised hepatitis C RNA testing and hepatitis C treatment in Australia, January 2013 – June 2018. Changes in testing and treatment levels associated with DAA availability were assessed in an autoregressive integrated moving average (ARIMA) statistical model, and the impact by 2030 of different levels of testing and treatment were estimated using a mathematical model.

**Major outcome measures:**

Hepatitis C prevalence among people who inject drugs; annual hepatitis C incidence relative to 2015 levels; projections for the hepatitis C care cascade in 2030.

**Results:**

The mean annual number of treatments initiated for people with hepatitis C increased from 6747 during 2013–2015 (before the introduction of DAAs) to 28 022 during 2016–18; the mean annual number of diagnostic RNA tests increased from 17 385 to 23 819. If current trends in testing and treatment continue (ie, 2018 testing numbers are maintained but treatment numbers decline by 50%), it is projected that by 2030 only 72% of infected people would be treated (by 2025 all people diagnosed with hepatitis C would be treated). The incidence of hepatitis C in 2030 would be 59% lower than in 2015, well short of the WHO target of an 80% reduction. The identification and testing of people exposed to hepatitis C must be increased by at least 50% for Australia to reach the WHO elimination targets.

**Conclusion:**

Hepatitis C elimination programs in Australia should focus on increasing testing rates and linkage with care to maintain adequate levels of treatment.

**The known:** Despite high initial uptake of hepatitis C treatment in Australia, it is uncertain whether the hepatitis C testing rate is sufficient to sustain the treatment uptake necessary for achieving the WHO hepatitis C elimination targets by 2030.

**The new:** Progress towards elimination in Australia was assessed in mathematical models incorporating data on hepatitis C testing and treatment from multiple national datasets. Australia is unlikely to meet the WHO targets unless the identification and testing of people exposed to hepatitis C is increased by 50%.

**The implications:** To maintain treatment levels adequate for achieving WHO elimination targets, hepatitis C programs in Australia should focus on increasing testing rates.

In 2015, the first World Health Organization viral hepatitis strategy^1^ set specific targets for the global elimination of hepatitis C as a public health threat by 2030: that the incidence of hepatitis C virus infections be reduced by 80% and hepatitis C‐related mortality by 65%. A major strategy for achieving these targets is to increase access to highly effective direct‐acting antiviral (DAA) treatments. The WHO viral hepatitis strategy includes two further targets for 2030: that 90% of cases of hepatitis C are diagnosed and that 80% of people with chronic hepatitis C are treated.^1^

Australia is one of only a few countries that have had unrestricted access to DAAs for several years. In 2016, the Australian government provided about $1.2 billion to fund DAA treatments over five years, so that every Australian with hepatitis C could receive treatment with low out‐of‐pocket cost to the patient ($39.50 per month or $6.40 per month for concession holders, and no out‐of‐pocket costs for Indigenous Australian patients).^2^ DAAs were made available without restrictions based on disease stage or risk behaviour, and re‐infected people were allowed to re‐commence treatment. In addition, broad access to treatment was ensured by policies that increased therapeutic capacity, including treatment of patients without significant liver disease by non‐specialist general practitioners in primary care.^3^

Modelling has indicated that Australia can meet the WHO 2030 elimination targets, provided treatment uptake can be sustained among people with advanced liver disease and people who inject drugs (the major group at risk of hepatitis C in Australia).^4^ During the first 15 months of DAA treatment availability (March 2016 to June 2017), 44 382 treatment courses were initiated in Australia,^5^ corresponding to 20% of the estimated 227 000 people with chronic hepatitis C in 2015.^6^ While this level of treatment uptake exceeded the estimated 12% of patients per year required to reach the elimination targets (4725 treatments among the estimated 40 000 infected people who inject drugs),^4^ early uptake reflected the availability of large numbers of patients waiting for DAAs to become accessible and easy‐to‐reach patients commencing DAA therapy. It is unclear whether high treatment rates can be sustained, and in some other countries they have declined.^7^, ^8^ The level of reduction that would put achievement of the elimination targets in Australia at risk is unclear.

To achieve the WHO hepatitis C elimination goals, the level of testing must be adequate. Modelling indicates that, if testing and engagement with care does not exceed pre‐2016 levels, the number of people treated will decline as the number of diagnosed and treatment‐ready patients declines.^9^ It is unclear whether the recent increase in testing in Australia is sufficient to sustain the treatment uptake level required for elimination.

In our study, we collated data on hepatitis C testing and treatment in Australia from several national datasets. Our key aims were to assess trends in hepatitis C testing and treatment after the introduction of DAAs, and to use mathematical modelling to assess how progress toward the 2030 WHO targets would be influenced by different testing and treatment levels.

## Methods

### Hepatitis C virus testing: Medicare Benefits Schedule rebates data

The presence of serum antibodies to the hepatitis C virus (HCV) indicates exposure to the virus; testing for HCV RNA (by polymerase chain reaction assay) is required to distinguish between an active infection and an earlier, resolved exposure.^10^ The costs of HCV antibody and RNA testing are borne in Australia by the government through the Medicare Benefits Scheme. As the Medical Benefits Schedule (MBS) item number for HCV antibody tests is shared with a number of other common blood tests, they cannot be specifically identified in MBS data. The MBS lists three categories for HCV RNA testing: qualitative testing to confirm active hepatitis C infection; quantitative testing in preparation for treatment; and qualitative testing to confirm treatment success. Our analysis focused on diagnostic RNA testing; quarterly time series data on the number of diagnostic RNA tests, by age category and sex, were obtained for the period 1 January 2013 to 30 June 2018 from Medicare item reports (http://medic​arest​atist​ics.human​servi​ces.gov.au/stati​stics/​mbs_item.jsp) (Supporting Information, part A).

### Hepatitis C treatment: Pharmaceutical Benefits Scheme data

Quarterly time series hepatitis C treatment prescription data for the period 1 January 2013 to 30 June 2018 were obtained from the Australian Pharmaceutical Benefits Scheme (PBS). Data were disaggregated by prescriber type (specialist, non‐specialist) and treatment regimen (DAA, pegylated interferon‐based) (Supporting Information, part A).

### Statistical analysis

Autoregressive integrated moving average (ARIMA) models^11^ were fitted to quarterly time series data for diagnostic RNA testing and treatment, including interruption terms from 1 April 2016 (quarter 2) to assess the impact of DAA treatment availability.

### Model projections

We applied the Burnet–Optima mathematical model of HCV transmission and liver disease progression and treatment used in our earlier studies of hepatitis C testing and treatment in Australia.^4^, ^9^ The cascade of care prior to the introduction of DAA therapy was fitted to the cascades of care for people who inject drugs (from a community‐based study^12^) and for other patients (from Kirby Institute surveillance reports^6^). The model‐calibrated proportions of HCV‐infected people who had undergone antibody or RNA testing in 2015 were respectively 75% and 61% of people who inject drugs, and 90% and 78% of other patients.^9^

For the period from 2016 (ie, period of unrestricted access to DAA therapy), four scenarios were simulated and compared:

- treatment numbers remain at pre‐2016 level (before 2016: pegylated interferon‐based therapy);
- initial increase in treatment numbers with introduction of DAAs, followed by decline from 2019 (ie, the current situation);
- treatment numbers in 2018 are maintained until 2030; and
- treatment numbers in 2018 are maintained until 2030, and testing (and diagnosis) of previously undiagnosed HCV‐infected people increases by 50% (Box 1).

Box 1 **Scenarios simulated in our mathematical model of hepatitis C virus transmission, liver disease progression, and treatment**

**Table:** 

Scenario	Testing inputs, 2018–2030	Treatment inputs, 2018–2030
No increase in treatment numbers	Mean pre‐2016 annual testing level maintained	Mean annual treatment numbers during 2013–2015 maintained, but treatment effectiveness changes in 2016 with introduction of DAAs
Initial increase in treatment numbers followed by decline (status quo)	2018 level maintained	2018: 20 000 (about twice during first half of 2018); 2019–2030: 10 000 per year
2018 treatment numbers maintained until 2030	2018 level maintained	2018–2030: 20 000 per year
2018 treatment numbers maintained until 2030 and testing numbers increase	50% higher than 2018 level	2019–2030: 20 000 per year

DAA = direct‐acting antiviral.

For each scenario, we assumed that treatment was not targeted to any subgroup of people with hepatitis C (ie, the chance of starting treatment is the same for people who inject drugs and for other patients).

The outcomes in each scenario were hepatitis C prevalence among people who inject drugs, annual hepatitis C incidence relative to 2015 levels, and the projected hepatitis C care cascade in 2030.

A Monte Carlo uncertainty analysis was conducted to obtain 95% confidence intervals (CIs) for model estimates: 100 random parameter sets were drawn, with parameters for the force of infection constant for people who inject drugs, disease progression and mortality rates, and the proportion of people spontaneously clearing infection selected randomly from their uncertainty bounds. A sensitivity analysis assessed the impact of data limitations, including testing in prisons not included in our model, lower RNA test positivity rate, and different levels of uptake of testing by people who inject drugs and other patients (Supporting Information, part B). Further model details have been reported elsewhere.^9^

Statistical analyses were undertaken in R 3.5.0 and modelling in Matlab 2018b.

### Ethics approval

Ethics approval was not required for our analysis of publicly available and non‐identifiable aggregate data.

## Results

### Testing and treatment time series

During 1 January 2013 – 31 December 2015, 20 240 hepatitis C treatments were initiated (mean, 6747 per year). During 1 January 2016 – 30 June 2018, 70 056 hepatitis C treatments were initiated (mean, 28 022 per year), including 67 180 instances of DAA therapy (96%); however, annual treatment numbers declined across this period (2016, 35 659; 2017, 24 769; first half of 2018, 9628) (Box 2).

During 1 January 2013 – 31 December 2015, 52 156 diagnostic HCV RNA tests were conducted (17 358 per year). During 1 January 2016 – 30 June 2018, 59 548 diagnostic HCV RNA tests were conducted (23 819 per year), including 32 519 tests (55%) for people in the 45–54 and 55–64‐year age groups (Box 2). Increases in overall diagnostic testing before and after the introduction of DAAs were much less pronounced than increases in treatment uptake: the annual number of treatments during 2016–18 was almost triple that of 2013–2015, while the annual number of diagnostic RNA tests increased by 37%.

Box 2 Hepatitis C diagnostic testing (Medicare Benefits Schedule items 69499 and 69500), by age category and sex, and treatment initiation (Pharmaceutical Benefits Scheme),* Australia, January 2013 – June 2018

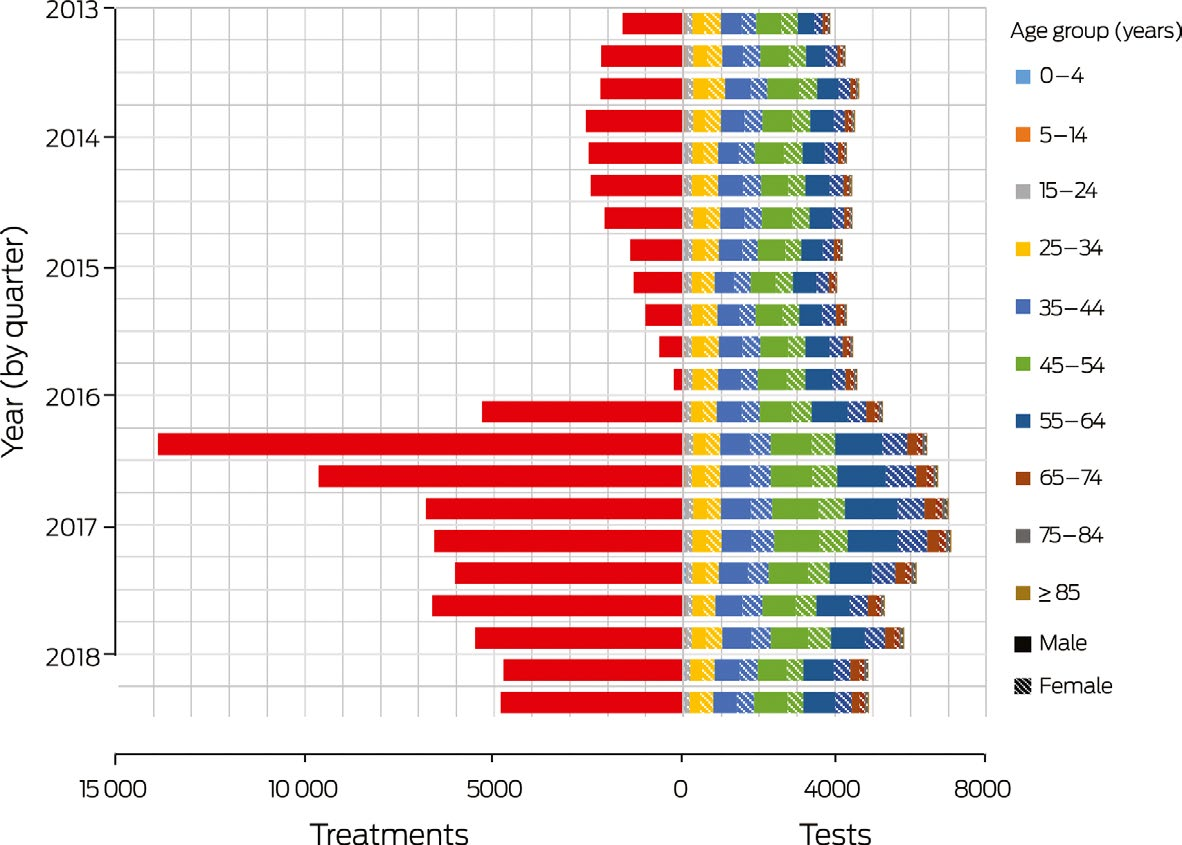

Changes in testing and treatment numbers in individual states and territories were similar to the national changes (Supporting Information, part C).

### Changes in treatment prescriber type

During 1 January 2016 – 30 June 2018, 35 434 of 67 393 treatments for which the prescriber type was identified (53%) were prescribed by specialists. The proportion of treatments prescribed by non‐specialists has increased since 2016: from 13 117 of 34 130 treatments (38%) in 2016 to 12 763 of 23 635 (54%) in 2017, and 6079 of 9628 treatments (63%) in the first half of 2018 (Box 3). This change in proportion was largely explained by declining numbers of treatments by specialists, as the numbers of treatments by non‐specialists were relatively stable.

Box 3 **Hepatitis C treatment initiation, Australia, January 2013 – June 2018, by prescriber type**

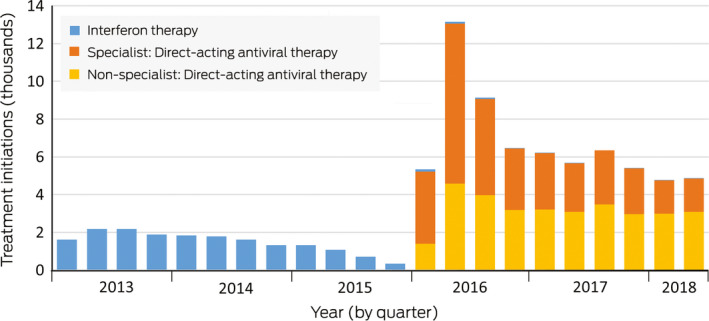

### Changes in diagnostic RNA testing following introduction of direct‐acting antivirals

The introduction of DAAs was associated with statistically significant increases of 1125 (95% CI, 359–1890) diagnostic RNA tests and 5647 (3661–7634) treatment initiations per quarter from the second quarter of 2016 (Supporting Information, part D); that is, a 26% increase on the background mean of 4323 (95% CI, 3541–5106) tests per quarter and a 315% increase on the background mean of 1794 treatment initiations per quarter.

DAA access was associated with statistically significant increases in testing for all age/sex groups, except for those aged 15–34 years and for women aged 45–54 years (Supporting Information, part D).

Data for the two non‐diagnostic RNA testing codes are provided in Supporting Information, part E.

### Model projections

Should present trends in testing and treatment continue (testing numbers maintained, treatment numbers declining to 10 000 per year by 2019), the model projected that by 2030 72% of people with hepatitis C would be treated (Box 4), the prevalence of hepatitis C among people who inject drugs would decline from 56% in 2015 to about 11%, and the incidence of new cases would be 59% lower than in 2015 (Box 5).

If current trends in testing level continue, the number of diagnosed but untreated people would approach zero by 2025 (data not shown). In the reduced and maintained treatment scenarios, an estimated 65 000 or 49 000 people would be undiagnosed or lost to follow‐up after a positive antibody test result in 2030 (Box 4). The epidemiologic outcomes for these two scenarios were similar: hepatitis C incidence and prevalence among people who inject drugs was slightly lower in the maintained treatment scenario because diagnosed people would be treated earlier, limiting the re‐infection rate.

In the increased testing scenario, the WHO treatment (80% of people with hepatitis C) and incidence targets (80% reduction) are achievable (Box 4, Box 5).

Box 4 **Model projections for the cascade of care in 2030 (with 95% confidence intervals), by simulation scenario**

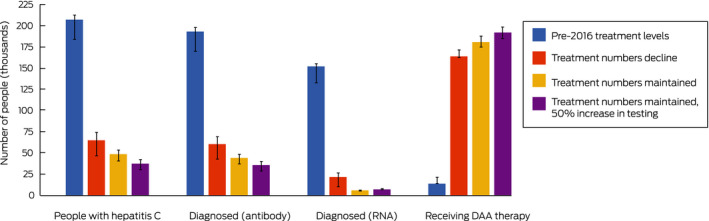

Box 5 **Model projections (with 95% confidence intervals) for hepatitis C incidence and prevalence among people who inject drugs in 2030, by simulation scenario**

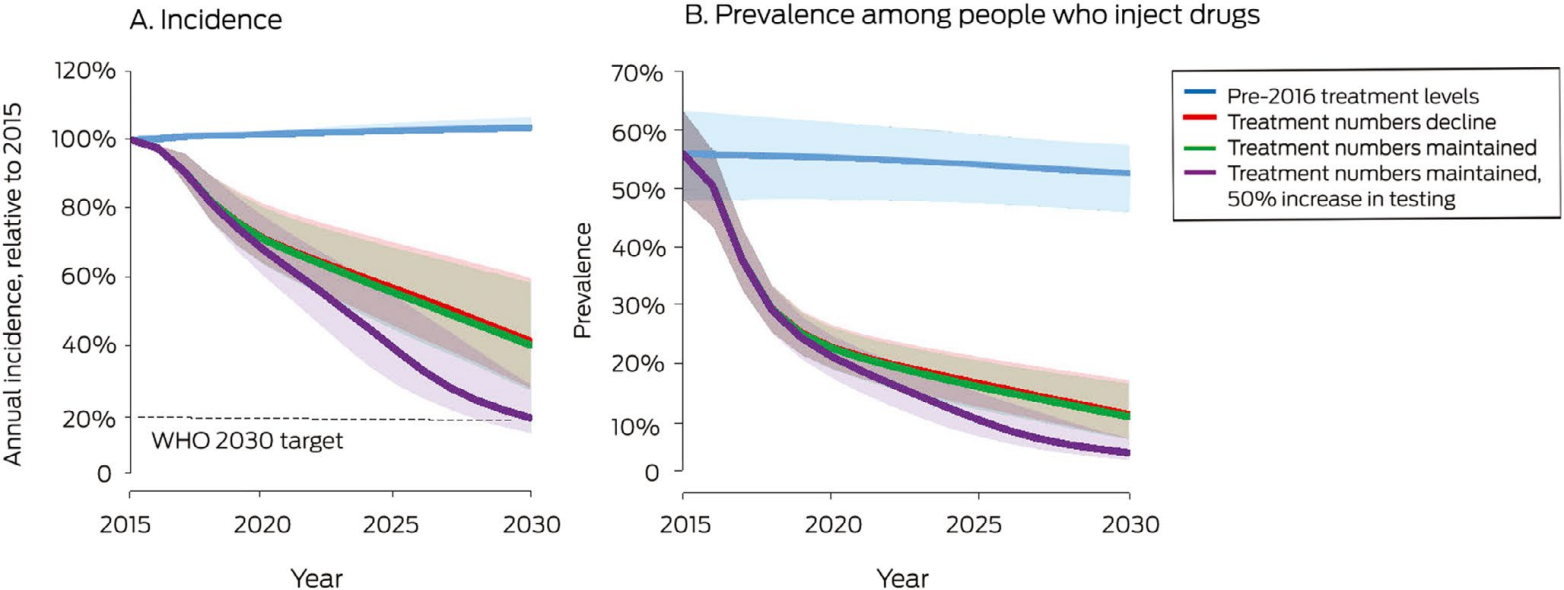

Our major findings were not altered by our sensitivity analyses (Supporting Information, part B).

## Discussion

After combining complete national datasets on hepatitis C RNA testing and treatment, we found that testing levels need to be increased to eliminate the disease in Australia. To reach the WHO elimination targets of 80% of people with hepatitis C being treated and incidence being reduced to 80% of the 2015 level by 2030, the number of hepatitis C‐exposed people identified and tested needs to be increased by at least 50%. Our finding contrasts with previous modelling which suggested that Australia was on track to achieving the 2030 elimination targets;^13^ however, the earlier projection was predicated on treatment numbers being maintained at current levels. In our study, we additionally modelled the hepatitis C care cascade, and found that treatment numbers will decline to fewer than required to meet the elimination targets unless testing is increased.

A key feature of our model is that people need to be diagnosed to receive treatment. That is, the annual number of people who commence treatment in each scenario is determined by the number of treatments available and the number of diagnosed people available for treatment initiation. Before DAAs were introduced, the number of tests exceeded that of available treatments, leading to an accumulation of diagnosed but untreated people; after their introduction, the number of treated people exceeded that of tested persons, and the number of diagnosed but untreated people gradually approached zero.

After the sharp initial increase in diagnostic RNA testing after the introduction of DAAs, the quarterly number of tests increased only slightly; the proportional increase was small compared with that in treatment numbers. This suggests that most treatments were prescribed to people who had been diagnosed for some time. People who have had hepatitis C longer are likely to have more advanced liver disease than those recently infected, and were more likely to be among the backlog of patients in specialist care that accumulated before DAAs became available (Box 2); hepatitis C treatment numbers had declined immediately before DAAs were introduced because specialists deferred treatment. The focus on this older cohort of people previously diagnosed by HCV antibody testing may partly explain why testing did not increase in younger age groups, as RNA testing was primarily used to confirm that patients still had active infections.

Hepatitis C testing trends indicated by MBS data are largely consistent with other Australian data. The number of hepatitis C diagnoses notified to the Australian National Notifiable Diseases Surveillance System has been stable since 2008–2017, but has declined among people aged 18–25 years.^14^ This is consistent with the modest overall increases in testing indicated by MBS data and the absence of increased testing in younger age groups.

If treatment is predominantly of people who have been diagnosed some time ago, some people with newly acquired infections may be missed. In Australia, people with newly acquired infections are likely to be people who inject drugs, as sharing injecting equipment is the major route for HCV transmission, and low treatment uptake in this group would have consequences for overall viral transmission. While data from the Australian Needle Syringe Program Survey indicate that the prevalence of recent hepatitis C treatment (during the past 12 months) among people who inject drugs has increased from less than 3% before DAAs to 36% in 2017,^15^ our findings suggest that a subgroup of people who inject drugs with newly acquired infections is being missed. It is of particular concern that there has been minimal, if any increase in RNA diagnostic testing among people under 35, particularly men. Two‐thirds of people who inject drugs in Australia are men, and about 20% are younger than 35 years old (re‐analysed data from the Illicit Drug Reporting System^16^). While only a minority of all people who inject drugs, younger men constitute an important subpopulation because of their increased susceptibility to new infection during the initial years of injecting.^17^ If Australia is to reach its hepatitis C elimination targets, younger people who inject drugs need to be targeted for testing and treatment.

Our modelling suggests that hepatitis C RNA testing in Australia would need to increase by at least 50% for the WHO elimination targets to be achieved. One approach to increasing the identification of hepatitis C‐exposed people would be to use rapid point‐of‐care antibody tests, reducing the need for multiple appointments to obtain a diagnosis.^18^ In Australia, targeted testing programs are being piloted, including rapid point‐of‐care saliva testing for hepatitis C antibodies at needle and syringe program sites.^19^ While point‐of‐care antibody and subsequent RNA testing may reduce the number of patient visits required for a diagnosis, the care cascade could be improved further by employing RNA tests as screening tools, particularly as their costs decline. Other methods for improving the care cascade include providing standard on‐site hepatitis C testing in primary and secondary enhanced needle and syringe programs, increasing standard testing in community mental health services, opt‐out testing for prisoners, and introducing mandatory reporting of hepatitis C testing as key performance indicators for opioid substitution therapy clinics and prisons.

The cost implications of a large increase in testing must also be considered. On the one hand, treating hepatitis C is cost‐effective in Australia because it averts liver disease and its associated health care costs,^20^ and the risk‐sharing agreement between the Australian government and originator pharmaceutical companies (an unlimited number of treatments between February 2016 and February 2021 at a cost of $1.2 billion) provides incentives for spending on diagnosis and engagement with care. However, the cost and effectiveness of scaling up interventions for improving diagnosis is unknown, as are future test positivity rates (a major driver of testing costs^21^). The most effective mix of interventions for improving targeted testing and engagement with care, and the expected cost and cost‐effectiveness of continuing to pursue the WHO elimination targets, remain to be determined.

### Limitations

We were unable to assess HCV antibody testing, meaning that the RNA testing numbers reflect the success of broader testing programs in identifying exposed people, and not necessarily the scaling up of antibody testing programs in Australia. Second, the entire (unlinked) PBS and MBS datasets were included in our analysis, including repeat tests for some people and tests with negative results. As more people are treated, the pool of antibody‐positive/RNA‐negative individuals is increasing, meaning that the proportion of positive RNA test results will decline over time, and the need for increased testing will be even greater than we have estimated. Third, inconsistencies in how health practitioners use MBS codes when ordering RNA tests for diagnostic purposes, treatment commencement, and treatment outcomes are recognised (Supporting Information, table 1). Fourth, prisoners are not eligible for MBS rebates, so that tests in prisons were not captured in our data. In 2018, there were about 29 000 sentenced prisoners with a median sentence term of 1.9 years,^22^ and HCV antibody prevalence in prisons was 31% in 2013;^23^ as we believe that 50% of prisoners have been tested for HCV antibody, we estimate that an additional 5300 tests (an increase of 10%) may have been undertaken during 2016–18, fewer than required to sufficiently increase overall testing levels by 50%. As the most comprehensive RNA testing dataset available, MBS data can, despite these limitations, provide reasonable estimates of overall changes in testing levels.

### Conclusion

To eliminate hepatitis C as a public health threat in Australia by 2030, hepatitis C elimination programs should maintain treatment uptake by focusing on increasing testing and linkage to care.

## Competing interests

Joseph Doyle, Margaret Hellard, and the Burnet Institute receive investigator‐initiated research funding from Gilead Sciences, Merck, AbbVie, and Bristol‐Myers Squibb. Joseph Doyle is an advisory board member for Gilead Sciences, AbbVie, Merck. Alexander Thompson is an advisory board member for Gilead Sciences, AbbVie, Bristol‐Myers Squibb, Merck, and Roche Diagnostics, and a speaker for Gilead, Merck, Bristol‐Myers Squibb, AbbVie, Roche Diagnostics. Nick Scott has received investigator‐initiated research funding from Gilead Sciences. Amanda Wade has received investigator‐initiated research funding from AbbVie. No pharmaceutical grants were received for this study.

## Supporting information

Supplementary materials
