# Supplementary material for: Australia needs to increase testing to achieve hepatitis C elimination
Source: Med J Aust. 2020 Mar 13;212(8):365–70. doi: 10.5694/mja2.50544 (PMC7317196; doi:10.5694/mja2.50544)
Supplement: Supplementary file 1 — Supplementary materials [file MJA2-212-365-s001.pdf]

The Medical Journal of Australia

Supporting Information

Supplementary materials

This appendix was part of the submitted manuscript and has been peer reviewed.
It is posted as supplied by the authors.

Appendix to: Scott N, Sacks-Davis R, Wade AJ, et al. Australia needs to increase testing to achieve hepatitis C elimination. *Med J Aust* 2020; doi: 10.5694/mja2.50544.

A. Data on testing and treatment

Hepatitis C testing data: rebates from Australia's Medicare Benefit Scheme (MBS)

The MBS covers hepatitis C antibody tests, qualitative polymerase chain reaction (PCR)-based RNA tests, and quantitative PCR-based RNA tests.

When tests are requested by service providers, laboratories bill the MBS using specific item numbers. The MBS item number for hepatitis C antibody tests is shared with a number of other common blood tests, meaning that hepatitis C antibody tests cannot be independently identified. Accordingly, only hepatitis C RNA testing could be assessed; as RNA testing is required for treatment, it may better reflect linkage with care than antibody testing alone. The number of hepatitis C RNA tests reflects a number of factors, including the coverage of hepatitis C antibody testing, retention in care following a positive antibody test result, monitoring during treatment, and testing to detect reinfection after treatment.

The MBS has three charge categories for hepatitis C RNA testing that can be accessed independently (table 1). Service providers specify whether the RNA test is qualitative to confirm active hepatitis C infection; quantitative in preparation for treatment; or qualitative to confirm treatment success. In practice, this is an imperfect classification. Sometimes service providers omit these details (in which case laboratories will allocate the test to one of the three item codes), and there is also a restriction of one diagnostic RNA test rebate per person per year, which can lead to service providers requesting alternative codes to avoid cost to the patient (table 1). Nevertheless, these data represent the only complete, aggregate measure of hepatitis C RNA testing in Australia.

Hepatitis C treatment data: prescriptions from the Pharmaceutical Benefits Scheme (PBS)

The PBS covers all hepatitis C prescription data for Australia, since it is a government funded subsidy scheme open to all Australian residents. Data were obtained only for initial prescriptions, rather than repeat prescriptions, meaning that our assessment included treatment initiation rather than treatment completion (treatment completion data are not available). Data were disaggregated by prescriber type and treatment regimen. Prescriber types were classified as specialist or non-specialist according to PBS definitions (specialist prescriber types included "gastroenterologist", "hepatologist" and "infectious diseases physician", while non-specialist prescriber types included "general practitioner", "addiction worker" and "other" [eg, physician registrars]). Individual prescriber types were assigned according to the prescriber's primary listed specialty and registered postcode within the PBS system. Treatments regimens were classified into two groups: interferon-based (peginterferon alpha 2a or alpha 2b ± ribavirin, boceprevir, telaprevir), and interferon-free direct acting antivirals (DAAs) (sofosbuvir, daclatasvir, sofosfubir/ledipasvir, sofosfubir/velpatasvir, paritaprevir/ritonavir/ombitasvir/dasabuvir ± ribavirin, grazoprevir/elbasvir), referred to hereafter as "DAAs". The prescription data did not contain any demographic or risk behaviour data, meaning that differences in treatment uptake by age, gender or other factors could not be assessed.

Table 1. Medicare Benefits Scheme (MBS) item number for polymerase chain reaction (PCR)-based hepatitis C RNA testing

Item number	MBS description	Interpretation	Comments and limitations
69499 or 69500	Detection of hepatitis C viral RNA if at least one of the following criteria is satisfied:<br>▪ the patient is hepatitis C seropositive;<br>▪ the patient's serological status is uncertain after testing;<br>▪ the test is performed for the purpose of:<br>‣ determining the hepatitis C status of an immunosuppressed or immunocompromised patient; or<br>‣ the detection of acute hepatitis C prior to seroconversion where considered necessary for the clinical management of the patient.	RNA testing of antibody-positive people to detect current infection	These codes reflect diagnostic testing among those with hepatitis C antibodies. Limitations: includes repeated tests for some individuals, and may underestimate the number of hepatitis C diagnostic tests due to some healthcare providers ordering viral load tests (below) to avoid cost to the patient.
69488 or 69489	Quantitation of hepatitis C RNA load in plasma or serum in:<br>▪ the pre-treatment evaluation, of a patient with chronic hepatitis C hepatitis, for antiviral therapy; or<br>▪ the assessment of efficacy of antiviral therapy for such a patient	Viral load testing in preparation for treatment (following Australian guidelines[1])	These codes reflect the number of treatments.<br>Limitations: includes some diagnostic tests, ordered to avoid costs to the patient, which may be negative or repeat tests for some individuals.
69445 or 69451	Detection of hepatitis C viral RNA in a patient undertaking antiviral therapy for chronic hepatitis C	Testing to confirm treatment success (sustained viral response after 12 weeks of treatment [SVR12])	As items 69499/69500 are restricted to one per person per year, qualitative hepatitis C RNA monitoring during treatment requires this separate item code.

B. Model sensitivity analysis

Some additional scenarios were projected to assess the importance of data limitations. We modelled incidence and prevalence among people who inject drugs if:

- there were 10% more tests, reflecting the number of tests occurring in prisons;
- there were only half as many tests, reflecting a situation in which the results of half the RNA tests were negative (50% RNA test positivity rate);
- treatment uptake among people who inject drugs was only half that of the rest of the population, compared with the baseline projection, which assumed equal treatment uptake regardless of injecting status.

The major conclusion of our study, that Australia should focus on increasing testing rates to achieve hepatitis C elimination, was not altered by the findings of our sensitivity analyses (figure 1).

The model sensitivities to structural and population parameters have also been examined extensively previously.[2] These analyses found, consistent with other models of hepatitis C transmission, the greatest sensitivities to be:

- if the length of injecting career in Australia were shorter than the estimated 17 years,[3] treatment-as-prevention would be less effective, and for a given number of treatments incidence would be reduced less;
- if there were fewer people who inject drugs in Australia than the estimated 93 000,[4] incidence reduction would be greater than estimated;
- if there were more people who inject drugs in Australia than the estimated 93 000,[4] incidence reduction would be less than estimated.

Figure 1. Model sensitivity analyses

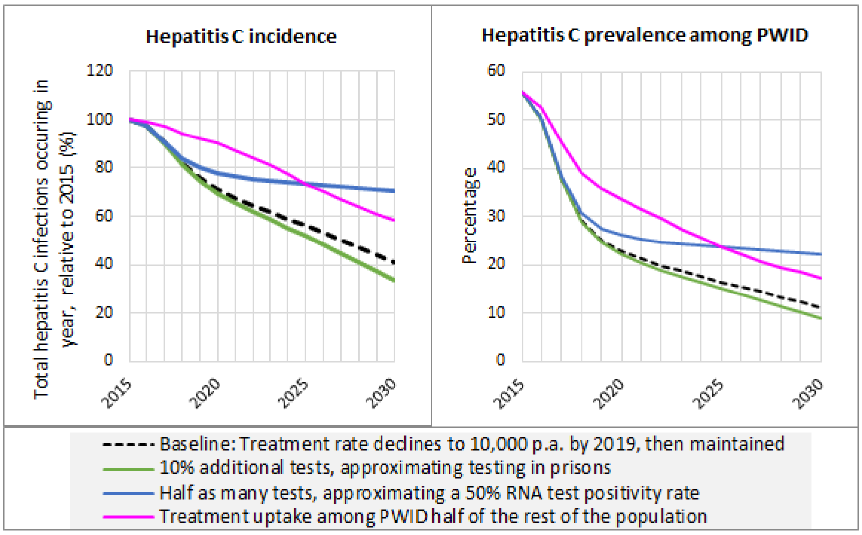

C. Hepatitis C diagnostic testing (Medicare Benefits Schedule items 69499 and 69500), by age category and sex, and treatment initiation (Pharmaceutical Benefits Scheme), January 2013 – June 2018, by state and territory

Figure 2. New South Wales

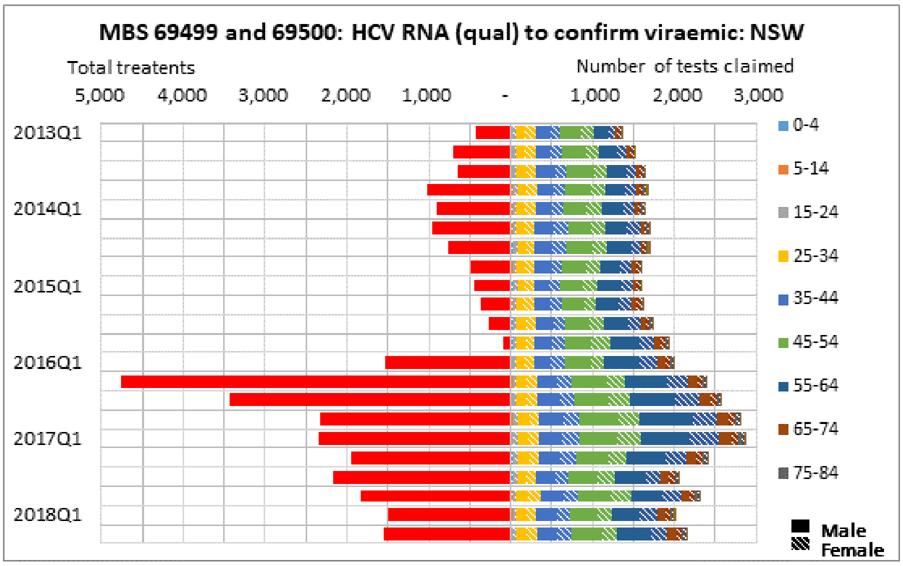

Figure 3. Victoria

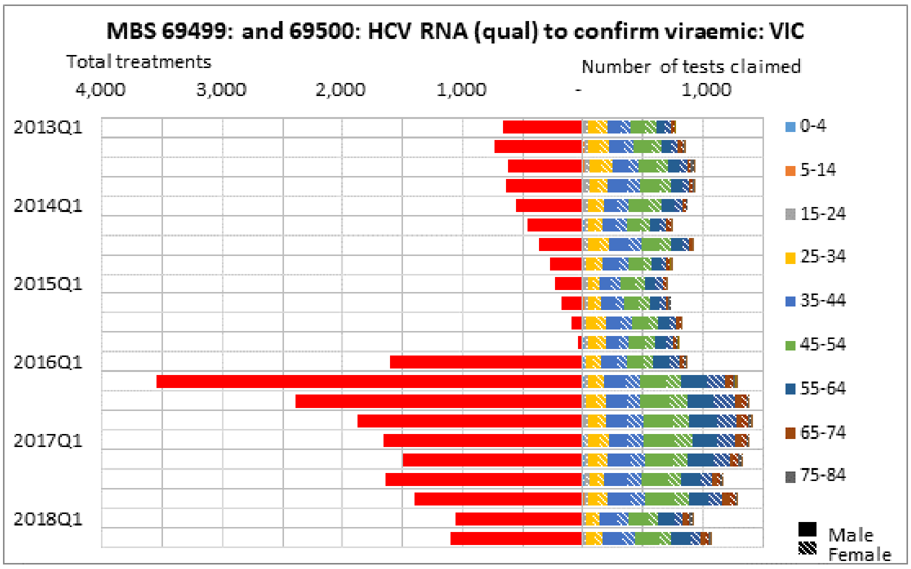

Figure 4. Queensland

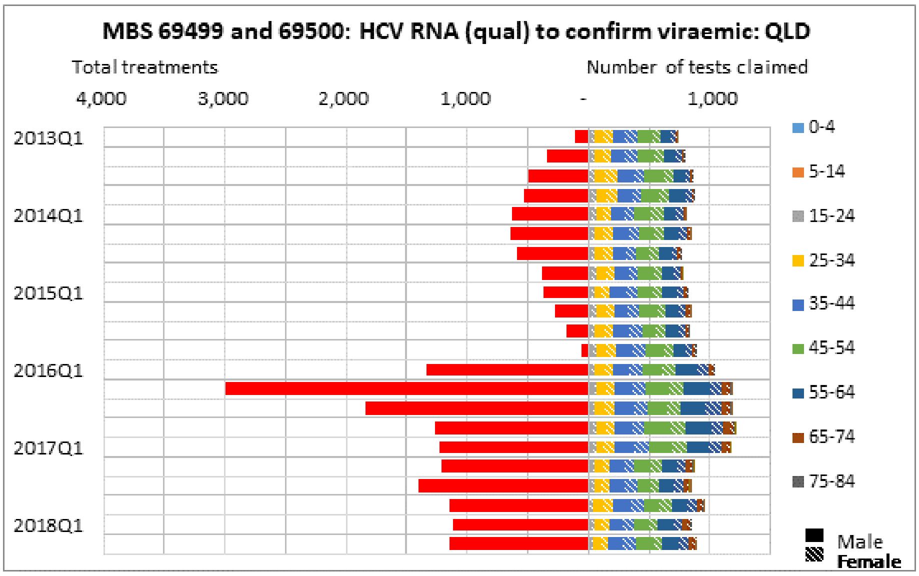

Figure 5. South Australia

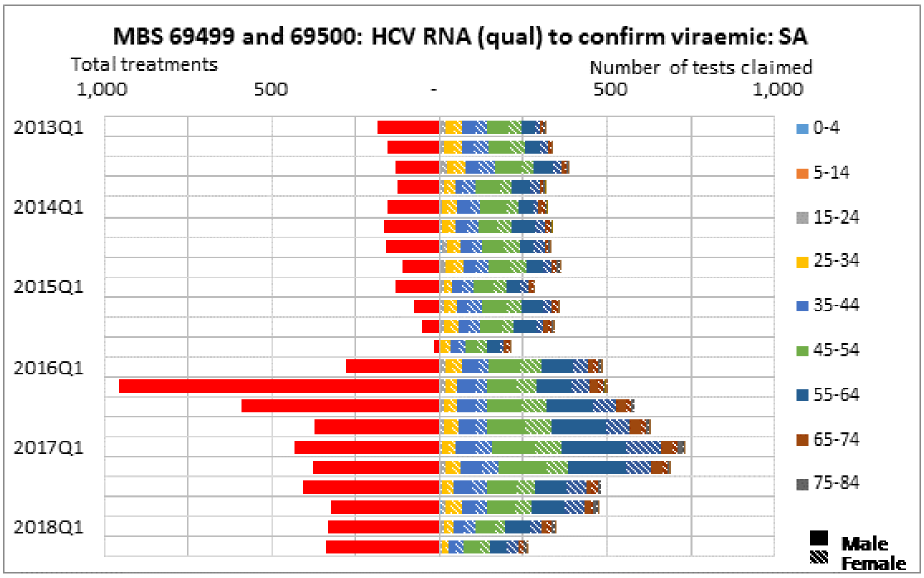

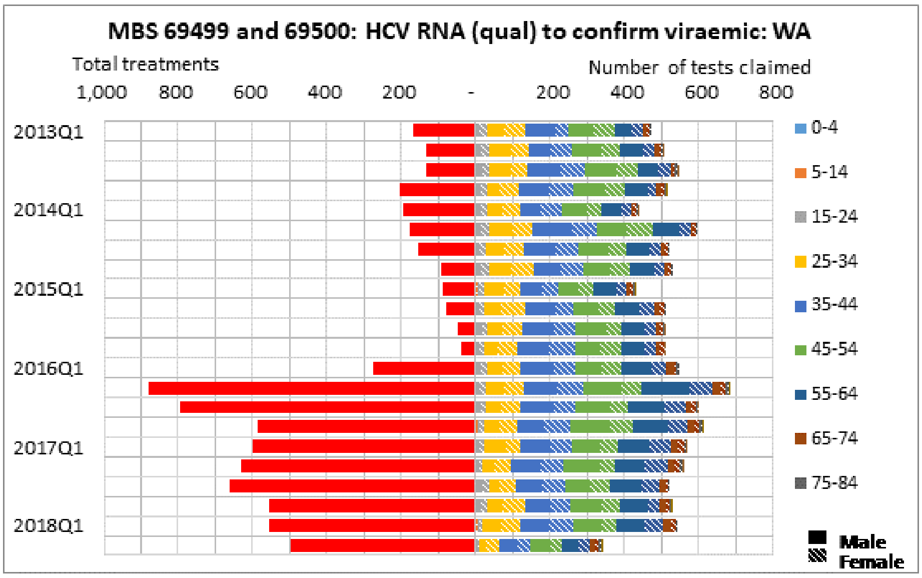

Figure 6. Western Australia

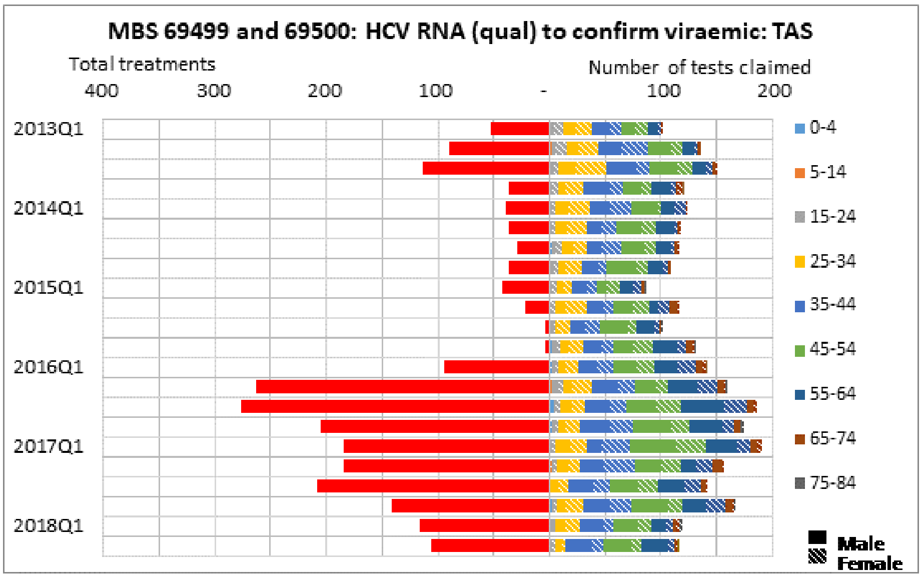

Figure 7. Tasmania

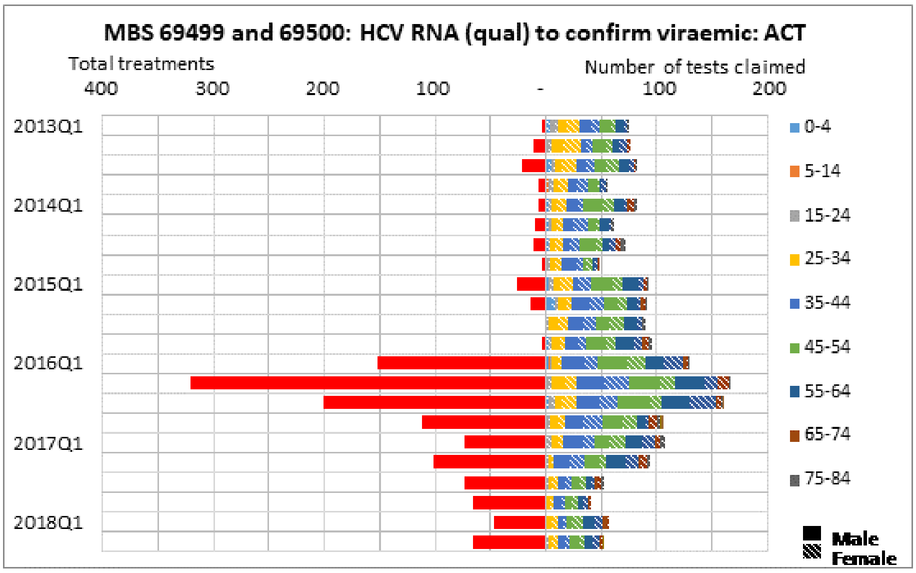

Figure 8. Australian Capital Territory

Figure 9. Northern Territory

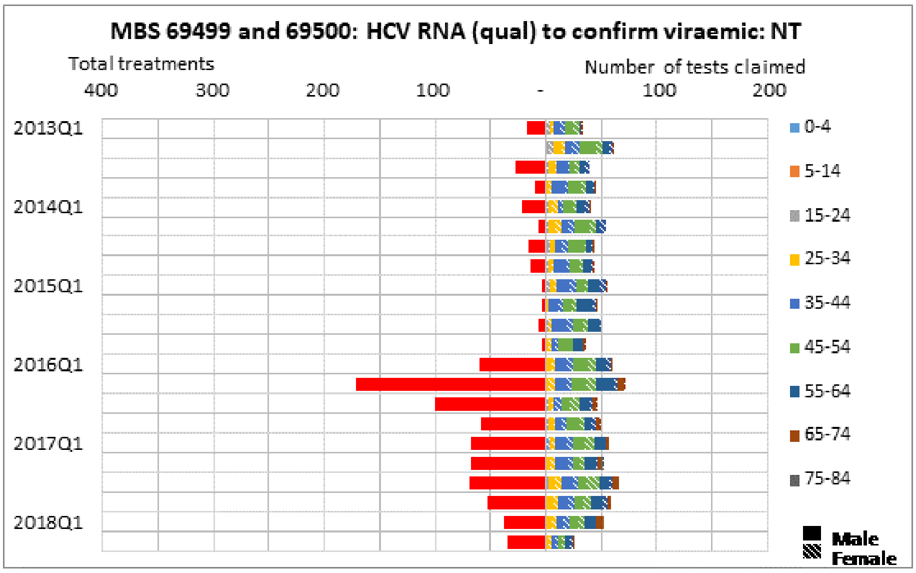

D. ARIMA models

The best-fit ARIMA model for the total diagnostic RNA tests (MBS item number 69499+69500) contained only a first degree auto-regressive term (table 2). The model was therefore:

$$y_{t+1} = \beta_1 DAA + \beta_2 y_t + \alpha + \epsilon_{t+1}$$

where y_{t+1} and y_t represent the observed number of tests in period *t+1* and *t* respectively, DAA is an interruption term (DAA=0 for t<2016Q2, DAA=1 for t>=2016Q2), ϵ_{t+1} is an error term for the period t+1, and β_1, β_2 and α are constants determined through regression.

The best-fit ARIMA model for the treatment numbers contained first and second degree auto-regressive terms. The model was therefore:

$$y_{t+1} = \beta_1 DAA + \beta_2 y_t + \beta_3 y_{t-1} + \alpha + \epsilon_{t+1}$$

Table 2. ARIMA model coefficients for diagnostic RNA testing and treatment initiation in Australia, January 2013 – June 2018, including an interruption term from April 2016 (the second quarter) for direct acting antiviral (DAA) availability

	p	d	q	DAA coefficient	Intercept/constant term (if applicable)	First degree autoregressive coefficient (if applicable)	Second degree autoregressive coefficient (if applicable)	Moving average coefficient (if applicable)
**Treatment**								
Total	2	0	0	5647 (3661–7634)***	1794 (511–3076)***	0.90 (0.53–1.28)***	–0.50 (–0.86 to –0.14)**	
**Testing**								
Total	1	0	0	1125 (359–1890)***	4323 (3541–5106)***	0.8 (0.56–1.04)***		
Male								
15–24	0	0	1	–12 (–22 to –1)†	80 (74–86)***			0.34 (–0.02–0.69)
25–34	0	0	1	–3 (–41 to 34)	335 (311–359)***			0.74 (0.45–1.04)***
35–44	0	0	1	115 (52–177)***	602 (563–641)***			0.45 (0.08–0.81)*
45–54	1	0	0	212 (71–354)**	737 (627–847)***	0.66 (0.33–0.98) ***		
55–64	2	0	0	197 (21–373)*	617 (374–861)***	1.29 (0.9–1.67) ***	–0.42 (–0.8 to –0.03)*	
65–74	1	0	0	51 (–7 to 109)	123 (47–200)**	0.89 (0.73–1.06) ***		
Female								
15–24‡	1	1	0	12 (–15 to 39)		–0.46 (–0.81 to –0.11)*		
25–34	2	0	0	17 (–55 to 90)	392 (265–519)***	0.44 (0.11–0.78)*	0.49 (0.13–0.84)**	
35–44	1	0	0	65 (19–112)**	449 (417–481)***	0.56 (0.22–0.91)**		
45–54	1	0	0	75 (–18 to 169)	468 (395–540)***	0.68 (0.38–0.98)***		
55–64	1	0	0	186 (44–327)*	313 (186–439)***	0.78 (0.53–1.04)***		
65–74	1	0	0	47 (15–79)**	81 (58–105)***	0.77 (0.49–1.06)***		

The forecast package in R[5] was used to determine suitable degrees of the auto-regressive (p), moving average (q) and difference (d) terms.

Statistically significant increases: * $P < 0.05$; ** $P < 0.01$; *** $P < 0.001$. Statistically significant decrease: † $P < 0.05$. Red: No statistically significant increase.

‡ No statistically significant increase with DAA introduction, but a longer term increase indicated by first degree difference term in the best-fit ARIMA model.

E. Additional MBS codes for HCV RNA testing

Viral load quantitative RNA testing more closely reflected treatment numbers (figure 10), which was expected because guidelines recommend this test pre-treatment.[1] RNA tests to confirm treatment success were lower than the treatment numbers and slightly time-lagged (figure 2). The time lag reflects treatment data being defined by treatment initiation, while testing to confirm treatment success is recommended 12 weeks after treatment completion (meaning 24–36 weeks after the treatment data point). The lower number of tests to confirm treatment success could explained by a number of factors, including a potentially high proportion of patients not returning for this test.[6-9]

Figure 10. Hepatitis C viral load testing in preparation for treatment (Medicare Benefits Schedule items 69488, 69489),[10] by age category and sex, and treatment initiation (Pharmaceutical Benefits Scheme), Jan 2013 – June 2018

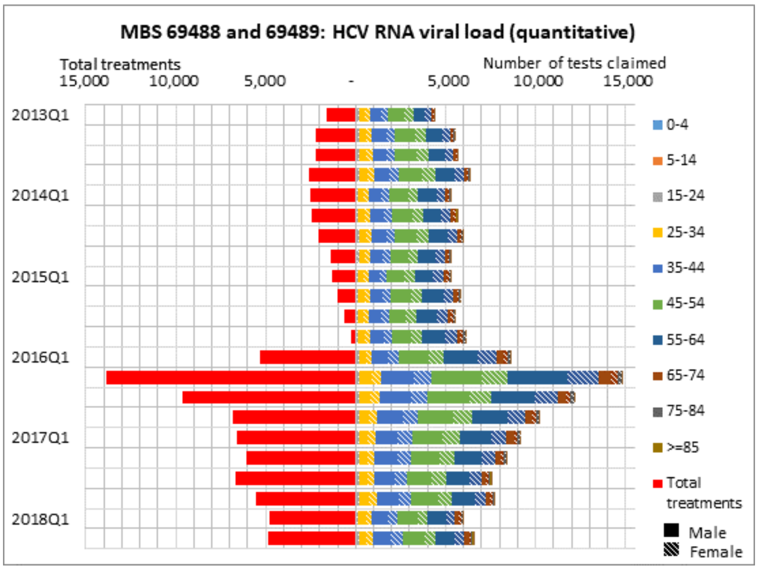

Figure 11. Hepatitis C viral load testing to confirm treatment success (Medicare Benefits Schedule items 69445, 69451),[10] by age category and sex, and treatment initiation (Pharmaceutical Benefits Scheme), Jan 2013 – June 2018

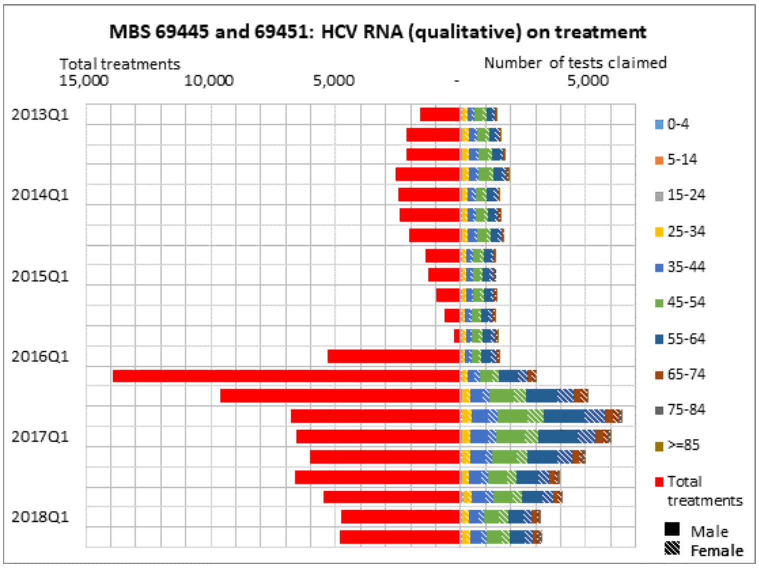

References

1 Australian Pharmaceutical Benefits Scheme. General statement for drugs for the treatment of hepatitis C. Updated Apr 2019. http://www.pbs.gov.au/info/healthpro/explanatory-notes/general-statement-hep-c (viewed Apr 2018).
2 Scott N, Doyle J, Wilson DP, et al. Reaching hepatitis C virus elimination targets requires health system interventions to enhance the care cascade. Int J Drug Policy 2017; 47: 107-116.
3 Fazito E, Cuchi P, Mahy M, Brown T. Analysis of duration of risk behaviour for key populations: a literature review. Sex Transm Infect 2012; 88 (Suppl 2): i24-i32.
4 Larney S, Hickman M, Guy R, et al. Estimating the number of people who inject drugs in Australia. BMC Public Health 2017; 17: 757.
5 Hyndman RJ, Khandakar Y. Automatic time series for forecasting: the forecast package for R: Monash University, Department of Econometrics and Business Statistics, 2007. http://webdoc.sub.gwdg.de/ebook/serien/e/monash_univ/wp6-07.pdf (viewed Jan 2020).
6 Kattakuzhy S, Gross C, Emmanuel B, et al. Expansion of treatment for hepatitis C virus infection by task shifting to community-based nonspecialist providers: a nonrandomized clinical trial. Ann Intern Med 2017; 167: 311-318.
7 Morris L, Smirnov A, Kvassay A, et al. Initial outcomes of integrated community-based hepatitis C treatment for people who inject drugs: findings from the Queensland Injectors' Health Network. Int J Drug Policy 2017;47: 216-220.
8 Read P, Lothian R, Chronister K, et al. Delivering direct acting antiviral therapy for hepatitis C to highly marginalised and current drug injecting populations in a targeted primary health care setting. Int J Drug Policy 2017; 47:209-215.
9 El-Akel W, El-Sayed M, El Kassas M, et al. National treatment programme of hepatitis C in Egypt: hepatitis C virus model of care. J Viral Hepat 2017; 24: 262-267.
10 Australian Department of Human Services. Medicare item reports. http://medicarestatistics.humanservices.gov.au/statistics/mbs_item.jsp (viewed Dec 2018).
